# Bacteremia by colistin-resistant *Acinetobacter baumannii* isolate: a case report

**DOI:** 10.1186/s13256-019-2062-3

**Published:** 2019-05-08

**Authors:** Julio Cesar Garcia Casallas, H. Robayo-Amortegui, Z. Corredor-Rozo, A. M. Carrasco-Márquez, Javier Escobar-Perez

**Affiliations:** 1 Facultad de Medicina, Evidence Therapeutic Group, Universidad de La Sabana, Campus Universitario, Km 7, Vía La Caro, Autopista Norte, Chía, Cundinamarca Colombia; 2 Clinical Pharmacology Department, Clínica Universidad de La Sabana, Chía, Cundinamarca Colombia; 3 Bacterial Molecular Genetics Laboratory, Universidad El Bosque, Bogotá DC, Colombia; 4 Critic Medicine and Intensive Care Resident, Universidad de La Sabana, Chía, Cundinamarca Colombia

**Keywords:** *Acinetobacter baumannii*, Colistin resistance, *bla*_OXA-23_, Bacteremia, Colombia

## Abstract

**Background:**

Carbapenem-resistant *Acinetobacter baumannii* infections are a major public health problem worldwide, requiring the use of “old” antibiotics such as polymyxin B and E (colistin). However, there is concern regarding the emergence of isolates resistant to these antibiotics.

**Case presentation:**

We report a case of a 64-year-old mestizo man hospitalized in an intensive care unit of a health institution in Colombia with identification and clinical and molecular typing of a colistin- and carbapenem-resistant *A. baumannii* isolate with mechanisms of resistance to colistin not previously reported, causing bacteremia.

**Conclusions:**

We have identified a strain of *A. baumannii* with mechanisms of resistance to colistin not previously reported in a patient with bacteremia who required treatment with multiple antibiotic schemes and had an adequate response.

## Introduction

The first global report on antibiotic resistance by the World Health Organization shows carbapenem resistance rates above 40% in *Acinetobacter baumannii*. Additionally, this bacterium is a leading cause of nosocomial infections, especially in intensive care units (ICUs), causing outbreaks with high mortality rates [1, 2]. *A. baumannii* has a natural resistance to some classes of antibiotics and an extraordinary ability to acquire resistance to others, including those regarded as last-line antibiotics, such as carbapenems. The most frequent carbapenemases in *A. baumannii* are the class D serine oxacillinases, which mainly belong to OXA-23. OXA-24, OXA-58, and OXA-143-like types begin mobilizing within plasmids or are inserted directly in chromosomes. In Colombia and other Latin American countries, the OXA-23 group-derived carbapenemases are the most prevalent. Carbapenem resistance has led to the need to use “old” antibiotics, such as fosfomycin and polymyxins (B and E). However, the use of these antibiotics has already caused the emergence of resistant isolates. In this scenario, without new antibiotics, the last option is treatment with combination therapies of two or more antibiotics [3]. This report describes a case of a patient with bacteremia caused by a pan-resistant *A. baumannii* isolate.

## Case presentation

On March 11, 2016, a 64-year-old mestizo man with a history of benign prostatic hyperplasia and use of an indwelling catheter presented to our emergency department with urinary retention. Cystoscopy revealed intravesical clots and obstructive bilobar prostate. Following the procedure, the patient exhibited signs of systemic inflammatory response syndrome and pathologic urinalysis. Therefore, antibiotic therapy with ampicillin/sulbactam was initiated without improvement in the clinical features. The urine culture report showed the presence of carbapenem-sensitive *Pseudomonas aeruginosa* and *Enterobacter cloacae*. Therefore, the therapy was subsequently escalated to meropenem (1 g every 8 h). The patient presented with clinical deterioration and ventilatory failure and was referred to the ICU for orotracheal intubation. He also developed cardiopulmonary arrest, which required basic and advanced resuscitation techniques for 11 min with subsequent sinus rhythm. On physical examination, the patient was under sedation, tachycardic, and hypothermic with evidence of purulent urethral discharge, and he required vasopressor support and sedoanalgesia. He had multiple organ dysfunctions due to urinary and pulmonary sepsis with the identification of carbapenem-resistant *Klebsiella pneumoniae* in the blood and lower respiratory tract secretions. The antibiotic therapy was adjusted to colistimethate (90,000 IU/kg) divided into three daily doses, doripenem (1 g every 8 h), and fosfomycin (4 g every 6 h). The patient initially progressed toward improvement, but 72 h later, he presented with new signs of inflammatory response. Therefore, a new blood culture was performed, revealing a carbapenem-resistant *A. baumannii* isolate (minimum inhibitory concentrations [MICs] ≥ 16, ≥ 16, and ≥ 8 μg/ml for imipenem, meropenem, and doripenem, respectively) that was also resistant to gentamicin (MIC ≥ 16 μg/ml), ciprofloxacin (MIC ≥ 4 μg/ml), and colistin (MIC 16 μg/ml) and sensitive only to tigecycline (MIC 1 μg/ml). Following this test, tigecycline administration was initiated (100 mg every 12 h), and colistimethate was suspended, with fosfomycin and doripenem continued until the completion of 14 days of treatment. Nevertheless, due to the persistent systemic inflammatory response on day 10, rifampicin (600 mg/day) and ertapenem (1 g every 12 h) were initiated for pharmacological synergism until day 14. With the prescribed antibiotic therapy, the patient improved in terms of infection, with an absence of fever and decreased leukocyte count. New blood and urine cultures were done, with negative results.

Molecular typing confirmed the genus and species of the isolate as *A. baumannii* (excluding other species of the *Acinetobacter* genus), as well as the presence of the *bla*_OXA-23_ gene (associated with carbapenem resistance). The isolate belonged to the sequence type (ST) 944, which has also been described in isolates identified in Russia, Italy, and the United States. Studies have identified intrahospital outbreaks of colistin-resistant bacteria with predominant genotype according to multilocus sequence typing was ST-258, ST11, ST273, and ST15 for *K. pneumoniae*; ISAba1 in *P. aeruginosa*, and pmrA1 and pmrB genotype ST94 for *A. baumannii* [4]*.* There are few reports on the resistance mechanisms associated with resistance to colistin in *A. baumannii*, the most important one being that related to loss in the production of lipid A of lipopolysaccharide. This loss has been associated with mutations in the *pmrAB* and *lpxACD* operons, *lpxK*, or the *IS*Aba11 insertion [5, 6]. The sequencing of these two operons in the *A. baumannii* isolate identified two nonsynonymous mutations (H31P and I215M) in the *lpxC* gene from the ATCC 19606 strain and another nonsynonymous mutation (A178G) in the *pmrC* gene from the ATCC 17978 strain [7–9]. None of these mutations has previously been reported in colistin-resistant strains. Although they have been found in the *A. baumannii* isolate, their possible association with resistance should be confirmed experimentally.

## Discussion

*A. baumannii* infections are a public health problem worldwide because there are fewer and fewer treatment options available. Multidrug-resistant (MDR) *A. baumannii* infection rates of up to 60% and mortality rates of 7% [10, 11] have been reported. One of the possible causes of this resistance is the indiscriminate use of antibiotics such as carbapenems, which are used in monotherapy in patients without comorbidities, resulting in increased resistance [12]. In the particular case of *A. baumannii* infection, the treatment options include the combination of carbapenems with tigecycline, aminoglycosides, and polymyxin B and E (colistin) [13]. Combination therapy including carbapenems aims for the “suicidal effect of the carbapenem,” and although it has been used in *K. pneumoniae,* it is also a treatment possibility for *A. baumannii* [14].

Regarding the use of polymyxin B or colistin, the former is usually preferred because its dosage is easier and more effective, the time to achieve optimal serum concentration is faster, there is less variability in pharmacokinetics, and no adjustment is required for renal failure. However, in our patient, the use of colistin was preferred due to the need to cover a pulmonary nidus and the possibility of inhaled administration [15–17]. Furthermore, fosfomycin is a broad-spectrum antibiotic that is especially used in combination therapy because it decreases the MIC of MDR strains and its use in monotherapy induces the development of resistance [18]. Currently, the clinical use of tigecycline in MDR infections is under discussion. Some meta-analyses suggest that it should be the treatment of choice in patients with carbapenemase-producing MDR gram-negative bacteria in combination with other classes of antibiotics. Synergism with polymyxins or carbapenems is not as clear as with other antibiotics such as fosfomycin or aminoglycosides [17]. Rifampicin was included in our patient’s regimen because this antibiotic, although effective in monotherapy, has a good synergistic activity with other antimicrobials, especially colistin, by permeabilizing the bacterial membrane and increasing the treatment effectiveness [19]. Although cefiderocol—a cephalosporin—is not a new class of antibiotic, it has a novel method of penetrating the tough outer membrane of gram-negative bacteria, including MDR strains. Attached to the drug’s main molecule is a siderophore, a compound secreted by bacteria to seek out iron, which bacteria need for survival, and transport it across cell membranes. The results of a phase 2 clinical trial suggest that a new treatment for patients with complicated urinary tract infections caused by MDR pathogens could be on the horizon [20].

## Conclusions

The emergence of colistin-resistant *A. baumannii* strains in the clinical setting, although still occasional, is increasing due to the growing use of this antibiotic. We have identified a strain of *A. baumannii* with mechanisms of resistance to colistin not previously reported in a patient with bacteremia, required treatment with multiple antibiotic schemes with adequate response. More studies are required to determine other possible genetic mechanisms associated with this resistance in order to develop a better approach to the management of patients with infections caused by this microorganism.
